# T‐tubule recovery after detubulation in isolated mouse cardiomyocytes

**DOI:** 10.14814/phy2.15779

**Published:** 2023-08-03

**Authors:** Greta Tamkus, Keita Uchida, Anatoli N. Lopatin

**Affiliations:** ^1^ Department of Molecular and Integrative Physiology University of Michigan Ann Arbor Michigan USA; ^2^ Present address: Department of Physiology Pennsylvania Muscle Institute, University of Pennsylvania, Perelman School of Medicine Philadelphia Pennsylvania USA; ^3^ Present address: John T. Milliken Department of Medicine Washington University School of Medicine St. Louis Missouri USA

**Keywords:** calcium, microtubule, osmotic stress, transverse‐axial tubule system, t‐tubule remodeling

## Abstract

Remodeling of cardiac t‐tubules in normal and pathophysiological conditions is an important process contributing to the functional performance of the heart. While it is well documented that deterioration of t‐tubule network associated with various pathological conditions can be reversed under certain conditions, the mechanistic understanding of the recovery process is essentially lacking. Accordingly, in this study we investigated some aspects of the recovery of t‐tubules after experimentally‐induced detubulation. T‐tubules of isolated mouse ventricular myocytes were first sealed using osmotic shock approach, and their recovery under various experimental conditions was then characterized using electrophysiologic and imaging techniques. The data show that t‐tubule recovery is a strongly temperature‐dependent process involving reopening of previously collapsed t‐tubular segments. T‐tubule recovery is slowed by (1) metabolic inhibition of cells, (2) reducing influx of extracellular Ca^2+^ as well as by (3) both stabilization and disruption of microtubules. Overall, the data show that t‐tubule recovery is a highly dynamic process involving several central intracellular structures and processes and lay the basis for more detailed investigations in this area.

## 1 INTRODUCTION

T‐tubules are surface membrane invaginations of cardiomyocytes that are critical for synchronized contraction. The structure of the t‐tubule network is dynamic with changes in the organization and density observed during development and disease (Dibb et al., 2021). During the early stages of heart failure, t‐tubules are remodeled which typically manifests in the form of t‐tubule dilations or overt loss of portions of the t‐tubule network (Lyon et al., 2009; Wagner et al., 2012; Wei et al., 2010). T‐tubule loss is associated with contractile defects and likely contributes to the depressed cardiac function observed in diseased hearts (Louch et al., 2010). The extent of pathological t‐tubule remodeling is strongly correlated with the regions of the myocardium that experience increased wall stress (Frisk et al., 2016). Alternatively, previous studies showed that the integrity of the cardiac t‐tubule network can be restored if the underlying mechanical stress is removed. For example, in a canine model, the ordered t‐tubule network structure lost during dyssynchronous heart failure was restored after cardiac resynchronization therapy (Li et al., 2015; Sachse et al., 2012). Additionally, in a rat model for heart failure induced by a mechanical load, complete mechanical unloading using heterotopic cardiac transplantation resulted in recovery of lost t‐tubules (Ibrahim et al., 2012). These studies raise the notion that cardiomyocytes have the intrinsic ability to restore lost t‐tubules, and presumably depressed cardiac function, if the underlying mechanical stress is removed. Unfortunately, specific mechanisms underlying the restoration of cardiac t‐tubules have not yet been investigated in detail and thus remain essentially unknown, largely due to the experimental difficulties associated with in vivo approaches employed in studying this process.

It has been known for quite some time that t‐tubular loss can be induced in cardiac myocytes in vitro using detubulation techniques involving osmotic stress (Kawai et al., 1999; Krolenko & Lucy, 2002; Moench et al., 2013) mimicking the effect of mechanical stress on t‐tubules observed in the whole heart models. Detubulation is characterized by formation of large t‐tubular dilations (vacuoles) separated by tightly constricted t‐tubules leading to electrical and diffusional disconnection between sealed t‐tubules and extracellular space (Uchida & Lopatin, 2018). Although it seemed that the loss of t‐tubules during experimental detubulation is essentially permanent, a potential recovery of t‐tubules following osmotic detubulation has never been investigated in detail. In this study we show that cardiac t‐tubules demonstrate a clear time‐dependent recovery following detubulation. In particular, the data show that the recovery process is highly dependent on the temperature, calcium entry during detubulation and microtubule dynamics. In conclusion, we present an easily accessible in vitro approach to study t‐tubule restoration following osmotic detubulation.

## 2 MATERIALS AND METHODS

### 2.1 Solutions

Modified Tyrode (Tyr) solution, myocyte storage solution (C solution), and hyposmotic Tyrode (0.6 Na) solution were prepared as previously described (Uchida et al., 2016). Formamide solution (1.5 M) was prepared in C solution.

### 2.2 Chemicals

Carbonyl cyanide‐p‐trifluoromethoxyphenylhydrazone (FCCP; Sigma, C2920), nicardipine hydrochloride (Nic; Sigma, N7510), colchicine (Col; Sigma, C9754), nocodazole (Noc; Sigma, M1404), taxol (Tax; Sigma, T7191), tetramethylrhodamine‐labeled 3 kDa dextran (TRITC; Fisher Scientific, D3307), Alexa Fluor 647 conjugated 10 kDa dextran (AF647; Fisher Scientific, D22914) and ethylene glycol‐bis (β‐aminoethyl ether)‐N,N,N′,N′‐tetraacetic acid (EGTA; Fisher Scientific, 409,911,000). Specific concentrations of agents are provided in the corresponding figure legends. 10 mM stock solution of colchicine was prepared in H_2_O and 20 mM stock solutions of taxol and nocodazole were prepared in DMSO.

### 2.3 Isolation of ventricular myocytes

Ventricular myocytes were isolated from the hearts of both male and female adult (ages ~3–5 months) wild‐type C57BL/6 mice using a published enzymatic isolation procedure (Moench et al., 2013) and were kept in C solution at room temperature until use.

### 2.4 Dextran trapping assay

Cells were detubulated either by application and removal of hyposmotic 0.6 Na or formamide solution as previously described (Kawai et al., 1999; Moench et al., 2013). Tetramethylrhodamine‐labeled 3 kDa dextran (TRITC) was introduced 2 to 5 min prior to the washout of hyposmotic or formamide solution, respectively. After removal of extracellular dextran, cells were incubated either on ice, at 25°C, or at 32°C for the specified duration. Afterwards, the cells were washed with ice cold C solution and kept on ice until imaging.

In a subset of experiments, two sequential formamide detubulations were performed. After the first detubulation in the presence of Alexa Fluor 647 conjugated dextran (AF647), extracellular dextran was removed, and cells were incubated either on ice or at 32°C as described. After the first incubation, cells were washed once with room temperature C solution and exposed to formamide solution again for the second detubulation in the presence of TRITC‐dextran. Afterwards, extracellular dextran was removed, and cells were kept on ice until imaging.

Double detubulation experiments were conducted using formamide since this approach seemed to result in a stronger detubulation.

### 2.5 Confocal imaging and analysis using FIJI (ImageJ)

Cells were imaged using an Olympus FV‐500 microscope (Microscopy and Image Analysis Laboratory, University of Michigan, Ann Arbor). Using FIJI/ImageJ software (https://imagej.nih.gov), images of cells were manually outlined and mean intracellular fluorescence of trapped dye per unit area was calculated. Background fluorescence observed in control cells was subtracted and relative fluorescence values were calculated by normalizing to the mean fluorescence of control cells undergoing a single detubulation.

To measure particle area and particle fluorescence intensity, a background subtraction was performed using a rolling ball radius of 20 pixels (~2.8 μm). All images within a dataset were uniformly thresholded with a constant value to produce binary masks of each image. The mask image was used to measure the particle area (A_part._; % area coverage of the total cell area) and to calculate the mean dextran fluorescence intensity within the particles (I_part._).

To analyze colocalization in FIJI, two channel confocal images of cardiomyocytes trapping TRITC‐dextran and AF647‐dextran were deconvolved using the DeconvolutionLab plugin and the Pearson's correlation coefficients were calculated within the intracellular space using the JaCoP plugin.

### 2.6 Dextran diffusion assay

A detailed description of this assay has been recently published (Uchida & Lopatin, 2018). Briefly, control or detubulated cardiomyocytes are placed in a rapid flow perfusion chamber and the t‐tubule network is locally filled with fluorescent dextran. Then, extracellular dextran is rapidly removed, and the t‐tubular fluorescence is monitored from a small intracellular spot using a wide‐field fluorescence microscope as the t‐tubule luminal dextran gradually diffuses out.

### 2.7 Electrophysiological measurements

Membrane capacitance and *I*_K1,tail_ currents were measured in whole‐cell voltage‐clamped cardiomyocytes as described in a previous study (Uchida et al., 2016). Images of patched cardiomyocytes were obtained using an MD500 microscope eyepiece camera and the AmScope 3.7 software (AmScope), and the cross‐sectional area was measured using ImageJ. Membrane capacitance is normalized to the cell cross‐sectional area and *I*_K1,tail_ currents were normalized to the *I*_K,end_ current.

### 2.8 Statistics

The data (mean ± SE) in each experimental series are from at least two heart preparations. Statistical significance was determined using a two‐sample two‐tailed unpaired *t*‐test assuming equal variances and considered significant if *p* < 0.05. In the figures, *, **, and *** correspond to *p* values of 0.05, 0.01, and 0.001, respectively.

## 3 RESULTS

### 3.1 Recovery of t‐tubules after detubulation

It has been previously demonstrated that fluorescent dextran in the extracellular solution will be trapped or “sealed” within t‐tubule lumens during osmotic detubulation (Brette et al., 2002). We have noticed in an earlier study (Moench et al., 2013) that the fluorescence of dextran trapped in sealed t‐tubules after hyposmotic detubulation slowly declined over time, but the details of this processes were not characterized. Original experiments were performed at room temperature (RT; ~19–22°C), so in this study we characterized the temperature dependence of the dynamics of dextran fluorescence decline after hyposmotic detubulation, in particular, with the goal of discriminating between purely physical (diffusion) or biological (t‐tubule remodeling) processes.

**FIGURE 1:**
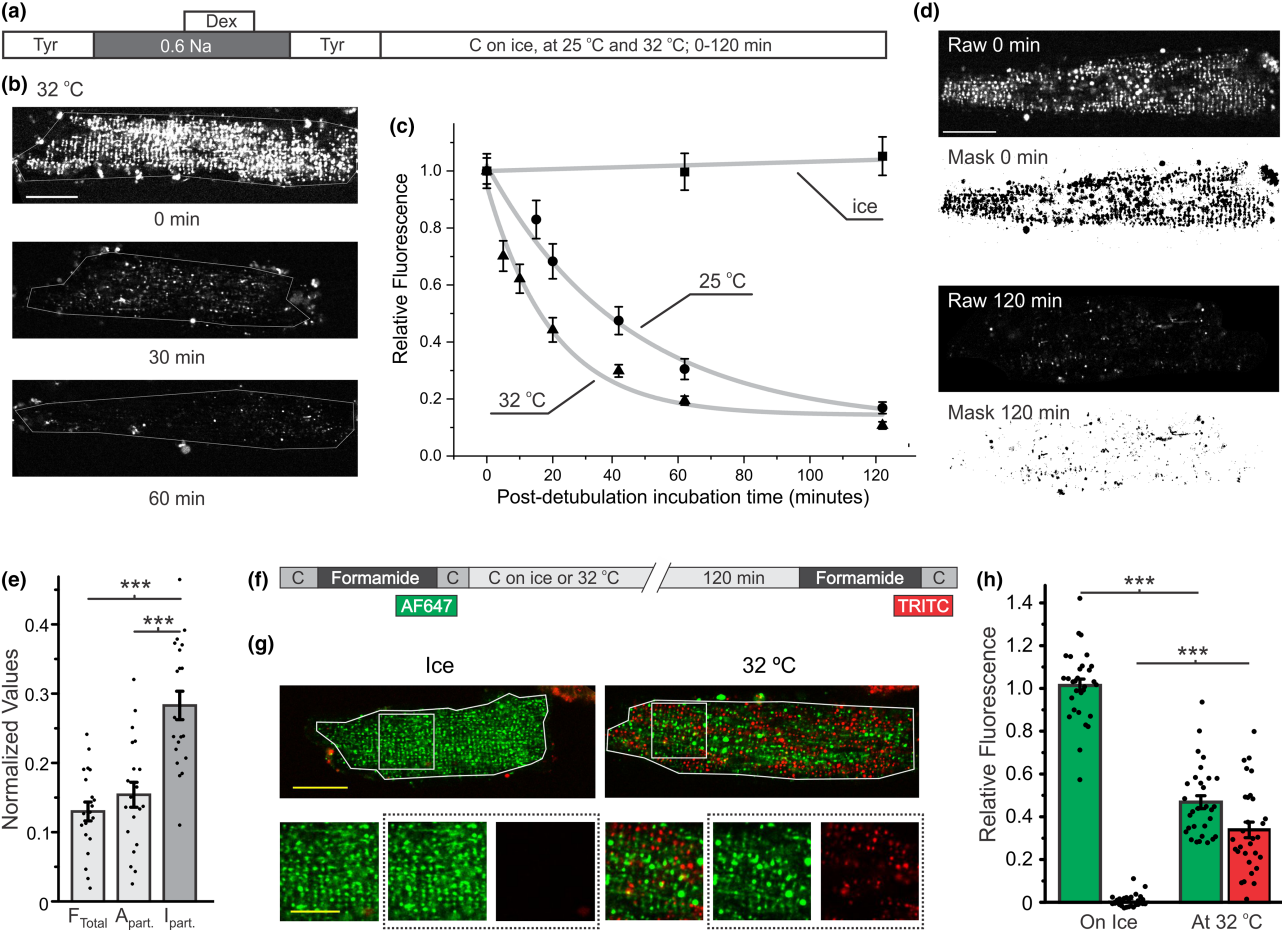
Dynamics of trapped dextran fluorescence after hyposmotic detubulation. (a) Protocol for characterizing the time and temperature dependences of trapped dextran fluorescence after detubulation. Cardiomyocytes were detubulated at room temperature in the presence of extracellular 3 kDa dextran by transient application of hyposmotic 0.6 Na Tyrode. Afterwards, cardiomyocytes were incubated at the indicated temperatures for 0–120 min before imaging. (b) Representative images of cardiomyocytes with trapped dextran following incubation at 32°C for 0, 30, or 60 min after detubulation with hyposmotic Tyrode. *Scale bar*: 20 μm. (c) Quantification of dextran fluorescence after detubulation. There were no significant changes in dextran fluorescence when cardiomyocytes were incubated on ice (linear fit; gray line). Time‐dependent decline in dextran fluorescence was characterized by single exponential approximation (gray lines): F = A*exp(*−t/τ*) *+ C*. At 25°C and 32°C the time course of dextran fluorescence was characterized by time constants *τ* ^h^_25_ = 42.7 ± 5.9 min and *τ* ^h^_32_ = 19.8 ± 3.8 min, respectively. Each point represents the average fluorescence from *n* = 20–30 cells from 2 to 3 separate preparations. (d) Representative images of cardiomyocytes with trapped dextran and the respective thresholded masks used to measure particle area (A_part._) and particle fluorescence intensity (I_part._). (e) Quantification of mean cell fluorescence (F_Total_), A_part.,_ and I_part._ at 120 min post‐hyposmotic detubulation normalized to 0 min. *N* = 20 cells from two separate preparations. (f) Protocol for experiments involving two sequential formamide detubulations. Cardiomyocytes were first detubulated to trap AF647‐(color coded green) dextran, incubated for 120 min either on ice or at 32°C, and then detubulated a second time to trap TRITC‐(color coded red) dextran. (g) Representative images of cardiomyocytes treated as described above. Yellow scale bars on the top and bottom panels represent 20 and 10 μm, respectively. The three bottom panels left to right show fluorescence of both dyes, fluorescence of AF647 only and fluorescence of TRITC only. (h) Quantification of the fluorescence of both dyes after the sequential detubulation. Relative fluorescence for each dye was calculated by normalizing to the average fluorescence of respective control cells that underwent a single detubulation. The first dye (AF647) showed a significant decline while the second dye (TRITC) showed a significant increase in fluorescence. *n* = 30 cells.

The fluorescence of trapped dextran was essentially unchanged over 2 h when the cells were placed on ice right after hyposmotic detubulation was performed at RT (Figure 1c), suggesting the preservation of sealed t‐tubules at this low temperature. This finding was essential in subsequent experiments to quantify the time dependence of remaining dextran fluorescence by transferring cells onto ice after a set time following detubulation (‘time freeze’).

The fluorescence of the trapped dextran declined slowly at 25°C and the time course of fluorescence decline could be characterized by a single exponential function with a time constant *τ* ^h^_25_ = 42.7 ± 5.9 min (Figure 1c; ‘^h^’‐hyposmotic). The decline in dextran fluorescence was significantly accelerated (relative to that at 25°C) when the samples were kept at 32°C after detubulation with *τ* ^h^_32_ = 19.8 ± 3.8 min.

Detubulation with formamide leads to a more complete detubulation than the 0.6 Na hyposmotic shock (Moench et al., 2013). To determine whether the magnitude of osmotic detubulation affects the kinetics of dextran fluorescence, its time course after formamide detubulation was quantified at 32°C. The data show that the dextran fluorescence declines much slower in cardiomyocytes following formamide detubulation than with hyposmotic shock. Specifically, the time constant of dextran fluorescence decline was ~threefold larger when compared to that obtained using hyposmotic detubulation: *τ* ^f^_32_ = 60.3 min (‘^f^’‐ formamide) versus *τ* ^h^_32_ = 19.8 min.

Since 3 kDa dextran is practically a membrane impermeable molecule, the most feasible pathway out of the sealed t‐tubules is by diffusion through constricted t‐tubule lumens rather than through internalization and secretion. This mechanism is supported by strong temperature dependence of the decline of total dextran fluorescence pointing to a gradual reopening, or recovery, of t‐tubules. Sealed t‐tubules and their recovery properties are expected to be highly heterogeneous, leading to significant differences in the time course of dextran fluorescence in individual t‐tubules. This, in turn, should lead to a difference in the rate of decline of the total area of sealed t‐tubules versus the fluorescence intensity of individual t‐tubules. Indeed, the data in Figure 1e show that 2 h after hyposmotic detubulation the relative change in the intensity of dextran fluorescence in individual t‐tubules (Particle Intensity; P_I_) is ~twofold smaller (larger remaining value) than that of their cross‐sectional area (P_Area_).

To confirm the above results and to further test the mechanism of t‐tubules recovery (in particular, to test whether t‐tubules retain their ability to seal again in response to further osmotic challenges) we performed a sequential formamide detubulation experiments as shown in Figure 1f–h. AF647‐dextran was trapped within t‐tubules during the first detubulation. As shown in Figure 1c, detubulated cardiomyocytes do not display a significant recovery of t‐tubules if kept on ice. Accordingly, when the detubulated cardiomyocytes remained on ice for 2 h before a second detubulation in the presence of TRITC‐dextran, only AF647 fluorescence could be observed in sealed t‐tubules. In contrast, when detubulated cells were incubated at 32°C for 2 h prior to the second detubulation, there was significant trapping of TRITC‐dextran (Figure 1g). Notably, the localization of the two dyes was essentially anticorrelated (Pearson's coefficient = 0.148 ± 0.012, *n* = 30 cells), suggesting a mixed population of t‐tubules: some t‐tubules remained sealed from the initial detubulation while other t‐tubules sufficiently recovered to allow TRITC‐dextran diffusion into the lumen and were subsequently resealed following the second detubulation (quantification in Figure 1h).

**FIGURE 2:**
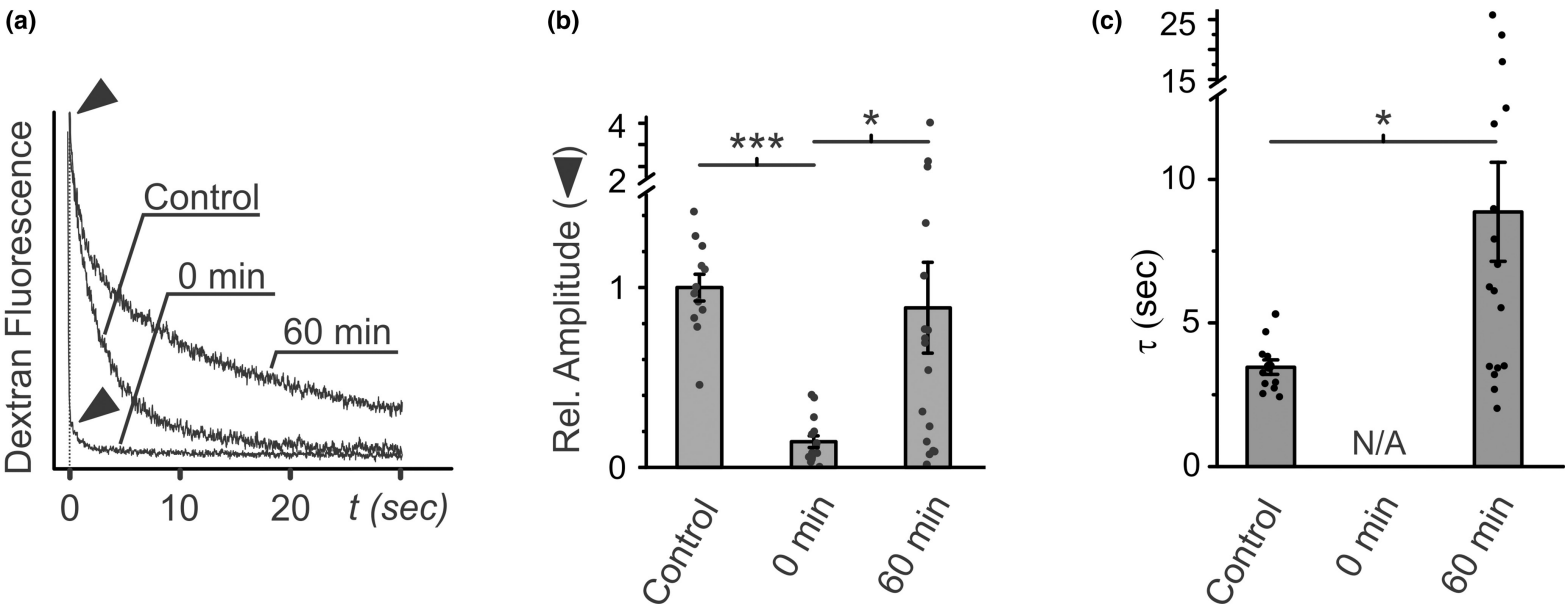
Recovery of diffusional properties of t‐tubules after hyposmotic detubulation. (a) Representative time courses of diffusion of t‐tubular dextran recorded in normal cells (Control), immediately after detubulation (0 min), or after detubulation followed by 1 h (60 min) incubation at 32°C. ▼‐ indicates the amplitude of t‐tubular dextran fluorescence. (b) Quantification of changes in fluorescence amplitude (reflects the volume of the t‐tubule network that is accessible to extracellular solution). Relative amplitude was not significantly different for control and 60 min conditions. (c) Quantification of changes in diffusion time constant of 3 kDa dextran (reflects diffusional accessibility of the t‐tubule network, in particular the magnitude of t‐tubular dilations and constrictions). *n* = 12, 15, and 17 cells from three separate preparations for control, 0 min, and 60 min, respectively.

To further quantify the accessibility of the t‐tubule network from the extracellular space after detubulation, we measured the kinetics of dextran diffusion out of sealed cardiac t‐tubules. In this assay, a patch pipette is used to locally apply fluorescent dextran to a small portion of the t‐tubule network and then the fluorescence of t‐tubular dextran is monitored as it diffuses out into the extracellular space (Uchida & Lopatin, 2018). The amplitude of dextran fluorescence reflects the volume of t‐tubules that are accessible to extracellular dextran while the kinetics of the fluorescence decline provides information about the diffusional accessibility of the t‐tubules. Dextran diffusion measurements were performed with normal non‐detubulated cells (control), cells immediately after hyposmotic detubulation (0 min) and detubulated cells after 60‐min incubation at 32°C. Figure 2a,c show that dextran fluorescence declines with a characteristic time constant of ~4 s in control cardiomyocytes, in agreement with past measurements (Uchida & Lopatin, 2018). In contrast, immediately after detubulation the time‐dependent component of fluorescence is essentially abolished indicating that the t‐tubules are no longer accessible to the extracellular solution consistent with t‐tubule sealing. However, after 60 min of incubation at 32°C, the time‐dependent component of fluorescence reappears (Figure 2a). Notably, while the amplitude of dextran fluorescence was essentially recovered after 60 min (Figure 2b), the time constant of fluorescence decline was nearly twofold larger, suggesting that dextran diffusion within t‐tubules remains restricted compared to that in control cells (Figure 2c).

**FIGURE 3:**
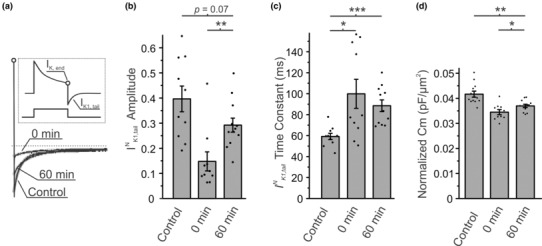
Electrophysiological characterization of diffusional properties of t‐tubules after hyposmotic detubulation. (a) Representative traces of *I*_K1,tail_ currents normalized to *I*_K,end_ (open circle) recorded from normal cells (Control), immediately after detubulation (0 min), or after detubulation followed by 1 h incubation at 32°C (60 min). *Inset*: the origin of *I*_K1,tail_ current. (b) Quantification of *I* ^N^_K1,tail_ current amplitudes. (c) Quantification of *I*_K1,tail_ time constant. (d) Quantification of membrane capacitance, C_m_. *n* = 10–12 cells, for each category.

To complement the results from the fluorescent dextran diffusion assay, the t‐tubule‐specific *I*_K1,tail_ current and membrane capacitance were measured under the same conditions as above. In brief, *I*_K1,tail_ current is an inward potassium current through *I*_K1_ channels induced by accumulation of t‐tubular K^+^ during preceding prolonged depolarization (Cheng et al., 2011; Clark et al., 2001) (Figure 3a (insert)), and characteristics of this current also reflect the diffusional properties of t‐tubules, now for K^+^ rather than dextran. Consistent with previous studies (Moench et al., 2013), the normalized *I*_K1,tail_ amplitude decreased ~2.7‐fold following detubulation, the time constant of the *I*_K1,tail_ increased by ~1.7‐fold and the normalized membrane capacitance, C_m_, was reduced by ~17% (Figure 3). After 60 min of incubation at 32°C, detubulated cardiomyocytes showed significant recovery of *I*_K1,tail_ amplitude and normalized C_m_. The *I*_K1,tail_ amplitude was not statistically different from that in control cells while the time constant of the *I*_K1,tail_ current and membrane capacitance did not fully recover to their original values after incubation.

Overall, these results strongly demonstrate that after osmotic detubulation sealed t‐tubules gradually reopen in a temperature‐dependent manner, although some defects in the diffusional properties of t‐tubule remain at least within the time scale of 1 h at 32°C.

### 3.2 Potential regulators of t‐tubule recovery after detubulation

**FIGURE 4:**
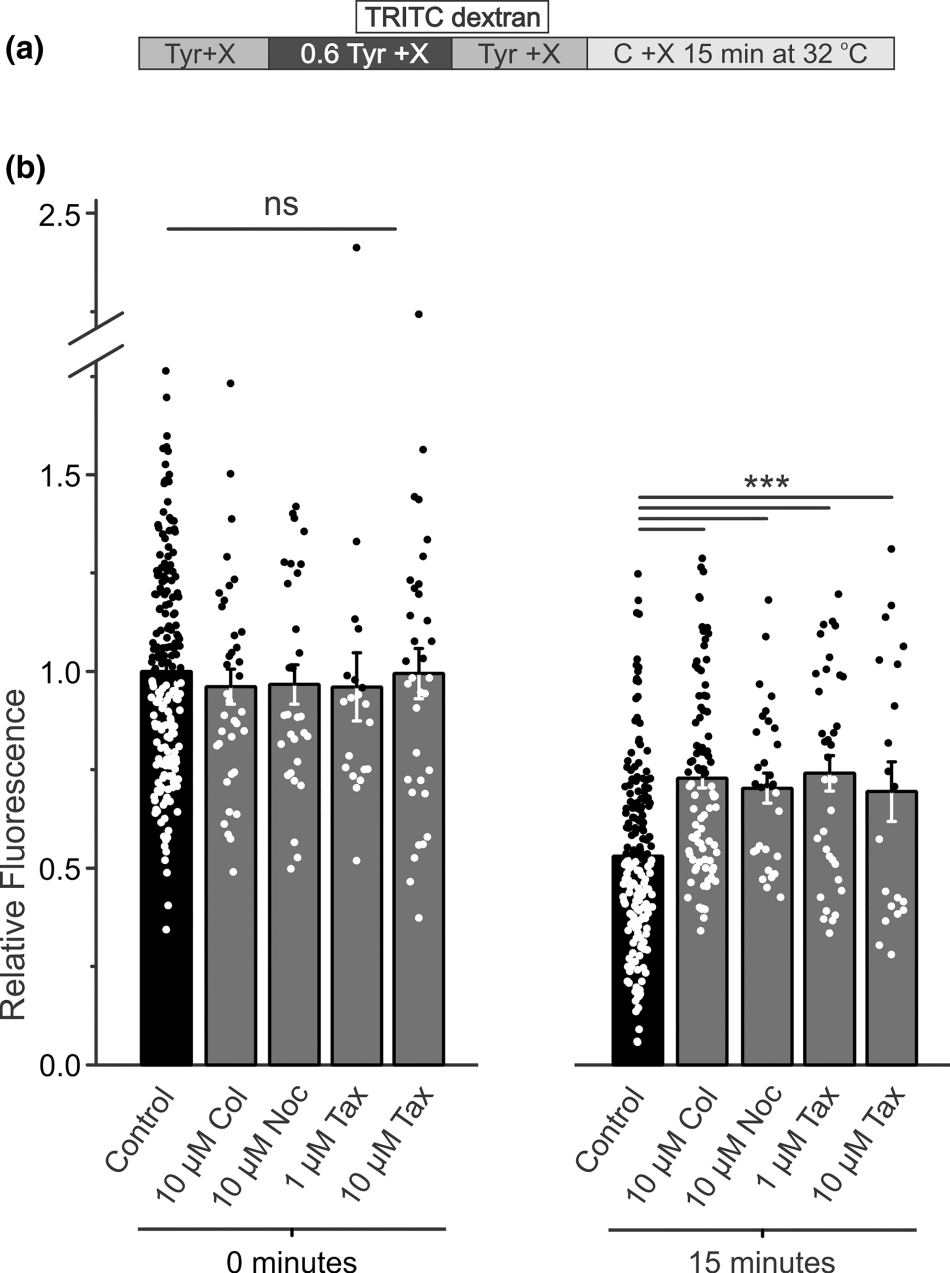
Effects of microtubule‐modifying agents. (a) Protocol for the application of pharmaceutical agents (X) using hyposmotically‐induced detubulation approach. (b) Effects of various microtubule‐modifying agents on the amount of trapped dextran immediately after detubulation (0 min) or 15 min after detubulation. Microtubule modifiers: disruptors colchicine (Col) and nocodazole (Noc), and stabilizer taxol (Tax). None of the agents had a significant effect on the initial amount of trapped dextran (0 min). However, both disruption and stabilization of microtubules led to an increased amount of trapped dextran after 15 min post‐detubulation. *n* = 200 (Control), 37–90 (10 μM Col), 20 (1 μM Tax), 34–35 (10 μM Tax), and 30 (10 μM Noc).

A significant slowing of t‐tubule recovery due to low temperature suggested that it may be mediated by changes in some intracellular processes and/or structures. For example, it is well established that microtubules can depolymerize at low temperatures (Bordas et al., 1983; Kirschner et al., 1974), which in turn have been implicated in regulation of many t‐tubular proteins (Hong et al., 2010; Zhang et al., 2014). Accordingly, to test the hypothesis that microtubules are involved in t‐tubule recovery, detubulation was performed in cardiomyocytes treated with microtubule‐depolymerizing agents and t‐tubule recovery was assessed after 15‐min incubation (this time provides the maximal dynamic range to identify treatments that either slow or accelerate t‐tubule recovery following osmotic detubulation) at 32°C (Figure 4a). The data show that treatment (involving pretreatment) with colchicine (a microtubule‐depolymerizing agent) had no effect on the magnitude of the initial hyposmotic detubulation but significantly slowed the t‐tubule recovery as evidenced by increased amount of trapped dextran 15 min after first detubulation (Figure 4b). Similar results were obtained in a complementary experiment with another microtubule‐depolymerizing agent, nocodazole (Figure 4b). Overall, these results suggested that an intact microtubule network promotes the recovery of t‐tubules following osmotic detubulation. We next tested the hypothesis that stabilization of microtubules with taxol treatment (including preincubation) would further promote t‐tubule recovery. Surprisingly, recovery of t‐tubules was also significantly slowed in the presence of taxol to a similar degree as that observed with microtubule disruption.

We showed in earlier studies that DMSO, a common solvent for various drugs, including those used in this study, has measurable effect on the magnitude of detubulation when present in the detubulating solution at relatively high, 1%, concentration (Uchida et al., 2016). The final concentration of DMSO used in this study is <=0.05% and no condition showed any changes in the initial hyposmotic detubulation (Figure 4b) and, therefore, potential effects of DMSO on detubulation can be safely dismissed. Additional experiments also showed that application of 0.05% DMSO after detubulation had no effect on relative fluorescence of trapped dextran during the recovery phase.

**FIGURE 5:**
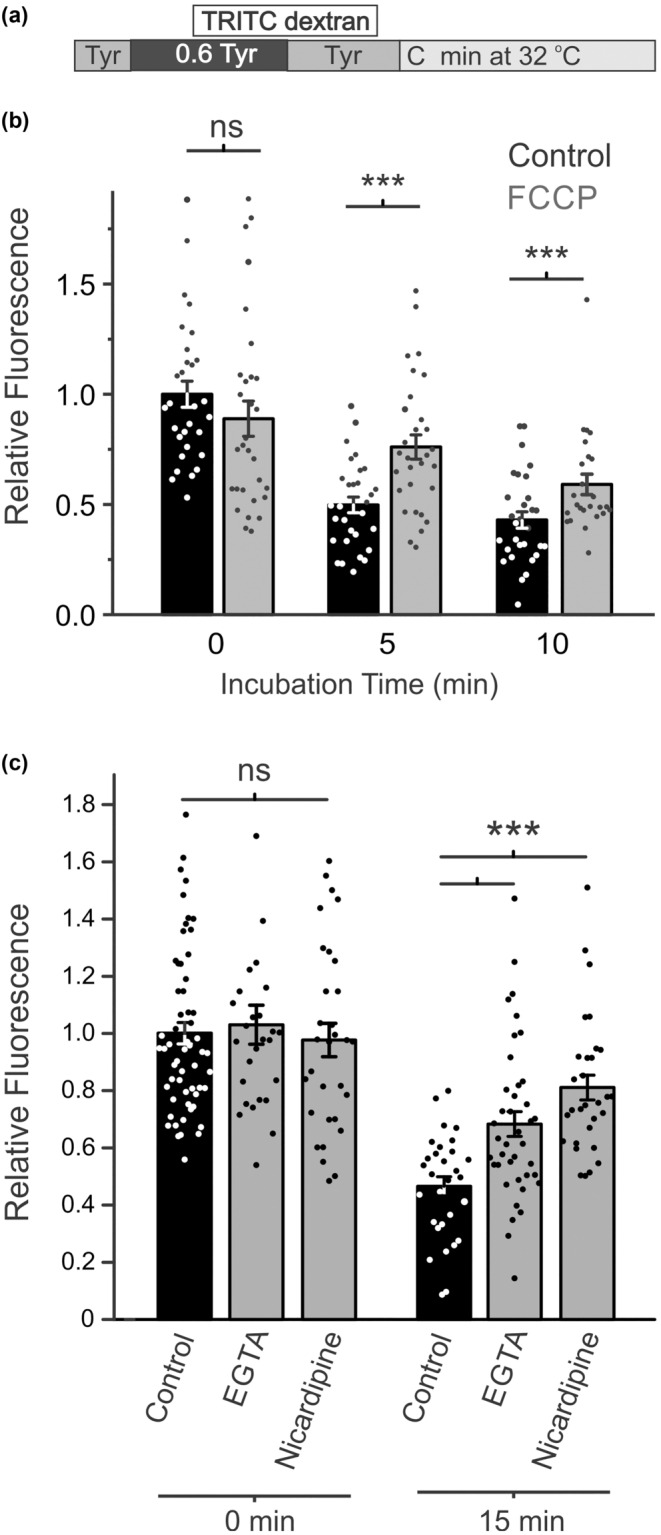
Effects of metabolic inhibition and Ca^2+^ manipulation. (a) Protocol for hyposmotic detubulation. (b) Quantification of the effects of FCCP (uncoupler of mitochondrial oxidative phosphorylation; 25 μM) on the post‐detubulation dextran fluorescence. FCCP was added immediately after detubulation. *n* = 25–30 cells. (c) Quantification of the effect of EGTA (1 mM) and nicardipine (10 μM) on the fluorescence of trapped dextran. EGTA and nicardipine were added during hyposmotic stress or after detubulation. Nicardipine was removed after incubation, but cells exposed to EGTA were maintained in EGTA containing solution to avoid unwanted effects due to calcium reintroduction. *n* = 20–30 cells.

In even earlier studies we showed that under certain experimental conditions, metabolic stress may have significant effects on the state of cardiac t‐tubules (Cheng et al., 2011). In this study we found that the recovery of t‐tubules after osmotic challenge can also depend on the metabolic state of cardiomyocytes. In particular, the data in Figure 5a show that inhibition of mitochondrial function with FCCP, a potent uncoupler of mitochondrial oxidative phosphorylation, leads to significant slowing of t‐tubule recovery, as evidenced by increased amount of trapped dextran. It should be noted that preincubation with FCCP leads to increased trapping of extracellular dextran during first detubulation (data not shown). Therefore, to isolate the effects of FCCP on t‐tubule recovery process, it was added to the incubation solution only after completion of first detubulation.

We previously reported that hyposmotic detubulation leads to a large calcium influx through L‐type Ca^2+^ channels in sealed t‐tubules (Moench & Lopatin, 2014). Here, we tested if this Ca^2+^ influx modulates the t‐tubule recovery process following hyposmotic detubulation. As shown in Figure 5c, neither chelating extracellular Ca^2+^ with EGTA nor blocking L‐type Ca^2+^ channels with nicardipine has any significant effect on the amount of trapped dextran after hyposmotic detubulation (Figure 5c; left). However, the amount of dextran retained in sealed t‐tubules after 15 min of incubation at 32°C in the presence of EGTA was significantly increased suggesting involvement of extracellular Ca^2+^ in the recovery process (Figure 5c; right). Similarly, blocking L‐type Ca^2+^ channels with nicardipine also led to slower recovery of t‐tubules, suggesting that the Ca^2+^ influx that occurs during detubulation may initiate some process that acts to restore t‐tubule structure.

## 4 DISCUSSION

### 4.1 Partial restoration of t‐tubule structure following osmotic detubulation

Osmotic detubulation of cardiac t‐tubules results in the rapid sealing of t‐tubule membranes and formation of dilated t‐tubular vacuoles that are diffusionally isolated from the extracellular solution and electrically uncoupled from the surface membrane. In this study, we show that (1) small dextrans trapped within sealed t‐tubules are released (Figure 1), (2) the t‐tubule luminal volume that is accessible from the extracellular space is increased (Figure 2), and (3) electrical coupling of t‐tubules with the surface membrane is recovered (Figure 3) in a time‐ and temperature‐dependent manner, strongly suggesting that following detubulation, t‐tubules gradually reopen. It also follows from this data that the apparently isolated t‐tubular vacuoles, as they may appear in the images of cardiomyocytes with trapped dextrans (Figure 1), remain interconnected by essentially collapsed (low volume) t‐tubular segments.

However, recovered t‐tubules still display significant functional differences compared to those in control, undisturbed cardiomyocytes, which likely reflect underlying structural defects. In particular, after 1 h of recovery, detubulated cardiomyocytes display a total dextran diffusion amplitude that is comparable to control cells (Figure 2) but have a membrane capacitance that remains only partially recovered (Figure 3b). The former suggests a similar t‐tubule luminal volume that can be filled with dextran compared to control cells while the latter reflects a reduction in the amount of t‐tubular membrane surface area. Combined, these results point to a t‐tubule network that may still be in a partially vacuolated or dilated state, consistent with the decreased t‐tubular surface area to volume ratio. Furthermore, the data in Figure 1d–h clearly demonstrate that not all t‐tubule segments recover at the same rate resulting in at least two populations of t‐tubule segments: those that have reopened and can be filled and detubulated a second time versus those that remain closed. The presence of t‐tubules that remain closed, even after a prolonged recovery time, suggests that the number of interconnections between neighboring t‐tubule segments are reduced during the recovery from detubulation and thereby increases the effective tortuosity of the t‐tubule network. The increased tortuosity combined with the remaining dilated/constricted t‐tubules results in a restriction of diffusion through the t‐tubule lumen (Figure 2c) (Uchida et al., 2020). Thus, during t‐tubular recovery, t‐tubular luminal content exchange is disrupted, which may have potential implications for ionic disturbances that may lead to an increased arrhythmogenic potential (Hong et al., 2014). It remains unclear if longer restoration times under current experimental conditions would lead to a more complete recovery of the t‐tubule network. In this regard, it is long known that even under culturing conditions long‐term incubation of isolated ventricular myocytes itself leads to the loss of t‐tubules (Lipp et al., 1996; Mitcheson et al., 1996).

### 4.2 Cellular mechanisms underlying t‐tubule recovery following osmotic detubulation

It is hard to imagine that a singular structure or process would be fully responsible for t‐tubule recovery after a global disruption of the t‐tubule network following osmotic detubulation. Importantly, the phenomena observed cannot be simply explained by slowed diffusion since t‐tubule recovery is completely halted for several hours on ice. Instead, it is highly likely that some, or many, major cellular systems would be involved. Accordingly, we identified several targets that modulate t‐tubule recovery following osmotic detubulation.

In particular, using a pharmacological approach, we found that microtubule network strongly affects t‐tubule recovery. In cardiomyocytes, microtubules form a complex cytoskeletal network composed of stabilized and dynamic microtubules and play many essential roles, including trafficking of various cargoes (Caporizzo et al., 2019). Although osmotic detubulation with formamide does not affect the overall organization or density of the microtubule network (Brette et al., 2002), it was surprising to find that t‐tubule recovery was strongly affected by microtubule targeting agents. Specifically, and unexpectedly, t‐tubule recovery was inhibited by both acute disruption of microtubules with colchicine or nocodazole and their stabilization with taxol (Figure 4b). These data suggest that microtubules should be in their dynamic state in order to take part in the t‐tubule recovery process.

T‐tubule formation involves a number of endocytic factors that stabilize tubulated membranes at the z‐disk (Hall et al., 2020), and several studies have demonstrated that the microtubule network plays a critical role in supporting endocytic tubules. For example, expression of Bin1, a BAR domain containing protein that is critical for t‐tubule biogenesis, in non‐cardiomyocytes induce the formation of long tubulated membranes (Lee et al., 2002). Bin1 interacts with microtubules through CLIP‐170 to direct the formation of these long tubules (Meunier et al., 2009). Experiments with nocodazole have demonstrated that these tubules become fragmented upon microtubule disruption but reform following microtubule repolymerization, suggesting that the formation and stabilization of Bin1 tubules are dependent on a dynamic microtubule network (Meunier et al., 2009). This Bin1–microtubule interaction is conserved in adult cardiomyocytes and directs the anterograde trafficking of Cav1.2 channels to the t‐tubular membrane in mouse cardiomyocytes (Hong et al., 2010). It is plausible that after t‐tubule disruption, dynamic microtubules may redirect new structural proteins to the sites of damage to restore t‐tubular structure. However, further experiments are necessary to elucidate the nature of this interaction.

While the effects of t‐tubule remodeling on Ca^2+^ handling are well known, the findings that chelation of extracellular Ca^2+^ with EGTA and block of L‐type Ca^2+^ channels with nicardipine significantly slowed t‐tubule recovery suggests that Ca^2+^ signaling pathways may be involved in initiating t‐tubule recovery. The identity of the effector of t‐tubule recovery downstream of this Ca^2+^ influx remains unknown. The finding that FCCP also inhibits t‐tubule recovery suggests that an ATP‐dependent process may also be involved. Further studies are required to elucidate the mechanism(s) regulating t‐tubule recovery following osmotic detubulation.

## AUTHOR CONTRIBUTIONS

Greta Tamkus and Keita Uchida conceived and designed research, performed experiments, analyzed data, interpreted results, prepared figures, drafted and edited manuscript. Anatoli N. Lopatin supervised research, analyzed data, interpreted results, prepared figures, and critically revised and edited manuscript.

## FUNDING INFORMATION

This work was supported by the National Institutes of Health Grants HL‐127023 (ANL) and T32‐GM‐008322 (KU) and American Heart Association Grant 17PRE33350049 (KU).

## CONFLICT OF INTEREST STATEMENT

No conflicts of interest, financial or otherwise, are declared by the authors.

## ETHICS STATEMENT

All experiments involving mice were carried out in accordance with the Guide for the Care and Use of Laboratory Animals (8th edition; The National Academic Press, Washington, DC) and protocols approved by the veterinary staff of the University Committee on Use and Care of Animals at the University of Michigan.
